# Metal deposition and shape reproduction at biological temperatures on cell-level samples

**DOI:** 10.1038/s41598-022-17562-9

**Published:** 2022-08-03

**Authors:** Kenshin Takemura, Taisei Motomura, Wataru Iwasaki, Naoki Matsuda

**Affiliations:** Sensing System Research Centre, The National Institute of Advanced Industrial Science and Technology (AIST), 807-1 Shuku-Machi, Tosu, Saga 841-0052 Japan

**Keywords:** Biotechnology, Biomimetics, Atomic force microscopy, Imaging techniques

## Abstract

The use of metal deposition has been limited to a limited number of applicable samples due to the increased temperature caused by accelerated electron impact on the substrate surface. The surfaces of various biological samples have a nanoscale structure with specific properties, which have been simulated in numerous studies. However, no examples of nano/microscale reproductions of biological surface features have used moulds. In this study, a mould that imitates the surface shape of a cellular-level biological material was fabricated, for the first time, and the shape was successfully reproduced using the mould. Al thin films were deposited on bovine sperm using magnetron sputtering without thermal denaturation with a cathode operating at a biological temperature. It is difficult to deposit films used as metal coatings on pre-treated biological materials at temperatures below 40 °C during evaporation. The Al thin film was peeled off and used as a mould to reproduce the shape of the sperm with high accuracy using a polymer. The results of this study represent a major innovation in reproducible biomimetic moulding technology, demonstrating biological temperature sputtering. We expect our non-destructive metal deposition and metal nano-moulding methods for biological samples to be the basis for the effective utilization of various biological structures.

## Introduction

Biological structures from micro to nano scale are known to exhibit a wide variety of structural properties and characteristics^1,2^. Biomimetics, which mimics and reproduces these functional properties of organisms or involves processes to synthesize useful materials, is an important field for innovatively functionalizing inorganic materials and process^3–5^. High-precision acquisition of structural information of biological materials focused on surface topography and detailed reproduction of the shapes would be an effective strategy for reproducing new functional materials^6,7^. Moreover, numerous functional materials with superhydrophobic, adhesive, or light-absorbing properties have already been developed based on the shape of biological samples^8–12^.

**Figure 1:**
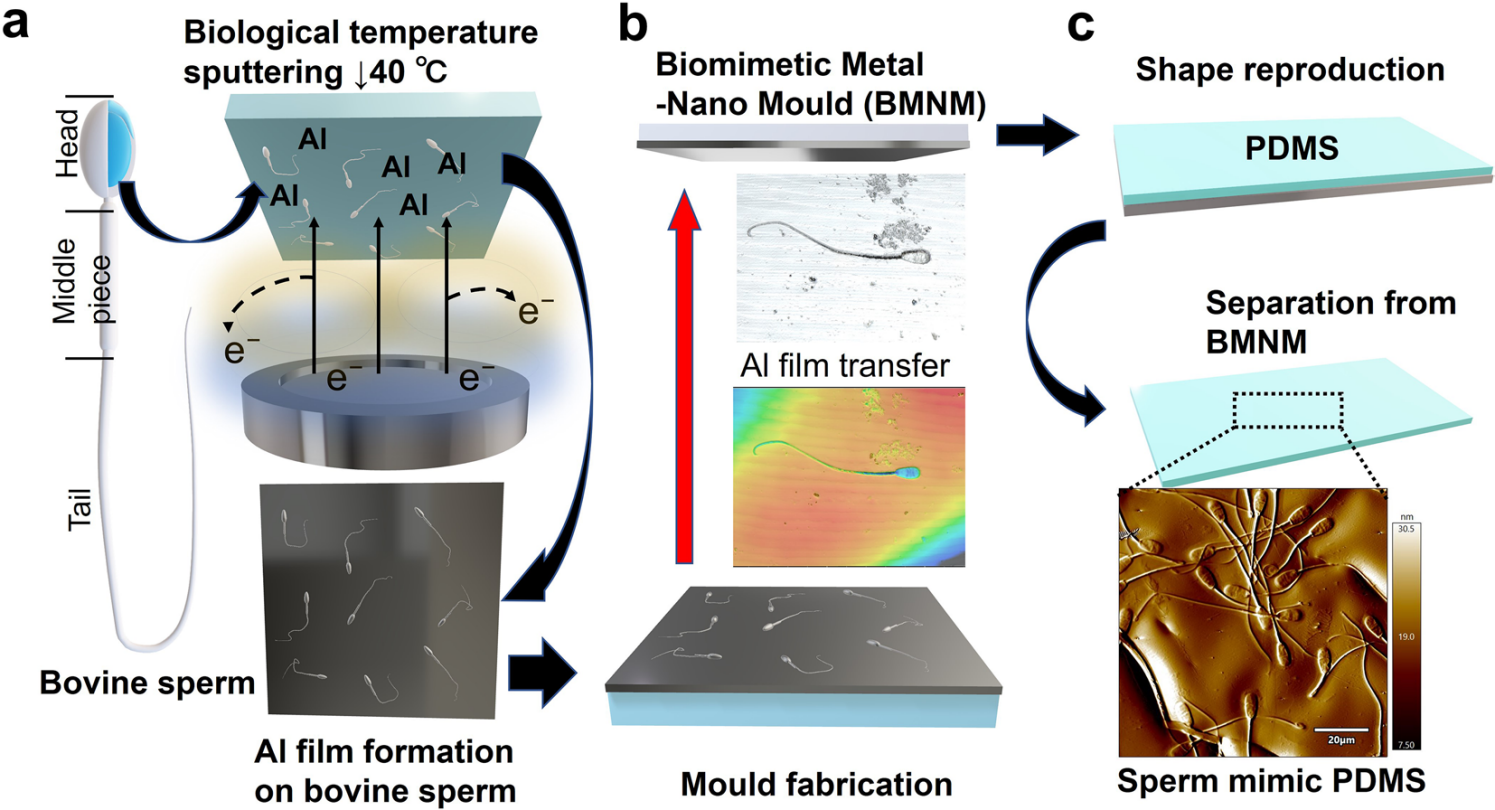
Schematic illustration of fabrication of biomimetic metal nano-mould (BMNM) and biomaterial shape reproduction. (**a**) Al thin film formed on adsorbed bovine sperm substrate at biological temperature. (**b**) Deposited Al thin film with preserved sperm shape. (**c**) Moulded dimethylpolysiloxane (polydimethylsiloxane, PDMS) reproduced shape of sperm.

Applying the surface structure of biological samples in material production essentially requires the acquisition of information through highly accurate observations with minimal damage. In particular, temperature changes should be controlled because they significantly affect the denaturation of protein structures^13–15^. Moulding the surface of biological materials is the simplest and most powerful means of exploiting their structural properties. However, no means existed to form metallic films conducive to moulding at low temperatures. In this study, we demonstrated non-heat shock metal sputtering of bovine sperm to fabricate a biomimetic metal nano-mould using confined high-temperature secondary electrons emitted from the target surface (Fig. 1a). The Al thin film, which was deposited in close contact with the sperm by sputtering, was peeled off from the substrate using adhesive tape (Fig. 1b).

Numerous dimples that maintained the shape of the sperm were formed on the Al thin film. These dimples were used as a mould into which dimethylpolysiloxane (polydimethylsiloxane, PDMS) was poured to reproduce the surface structure of the sperm on the polymer (Fig. 1c). Magnetron sputtering, which involves suppressing the temperature rise to near biological levels, was successfully used to deposit Al thin films without destroying the shape of the bovine sperm, which undergoes thermal denaturation above 42 °C. Furthermore, the thin film was peeled off the substrate and used as a mould to successfully reproduce the shape of a single-cell sample.

## Results

### Non-heat damage Al film coating on bovine sperm

Sperm in good condition were separated from the bovine semen preserved in a frozen state using a special microfluidic channel^16^. The adsorption of the separated sperm on the amino group-modified glass substrate was confirmed, and this adsorption was then compared with that of the sperm on a normal glass substrate using light microscopy (Extended Data Fig. 1). After adsorption, the sperm heads were immediately analysed using laser microscopy, and at approximately 10 µm, the diameter was consistent with that from data reported in a previous paper^17^.

**Figure 2:**
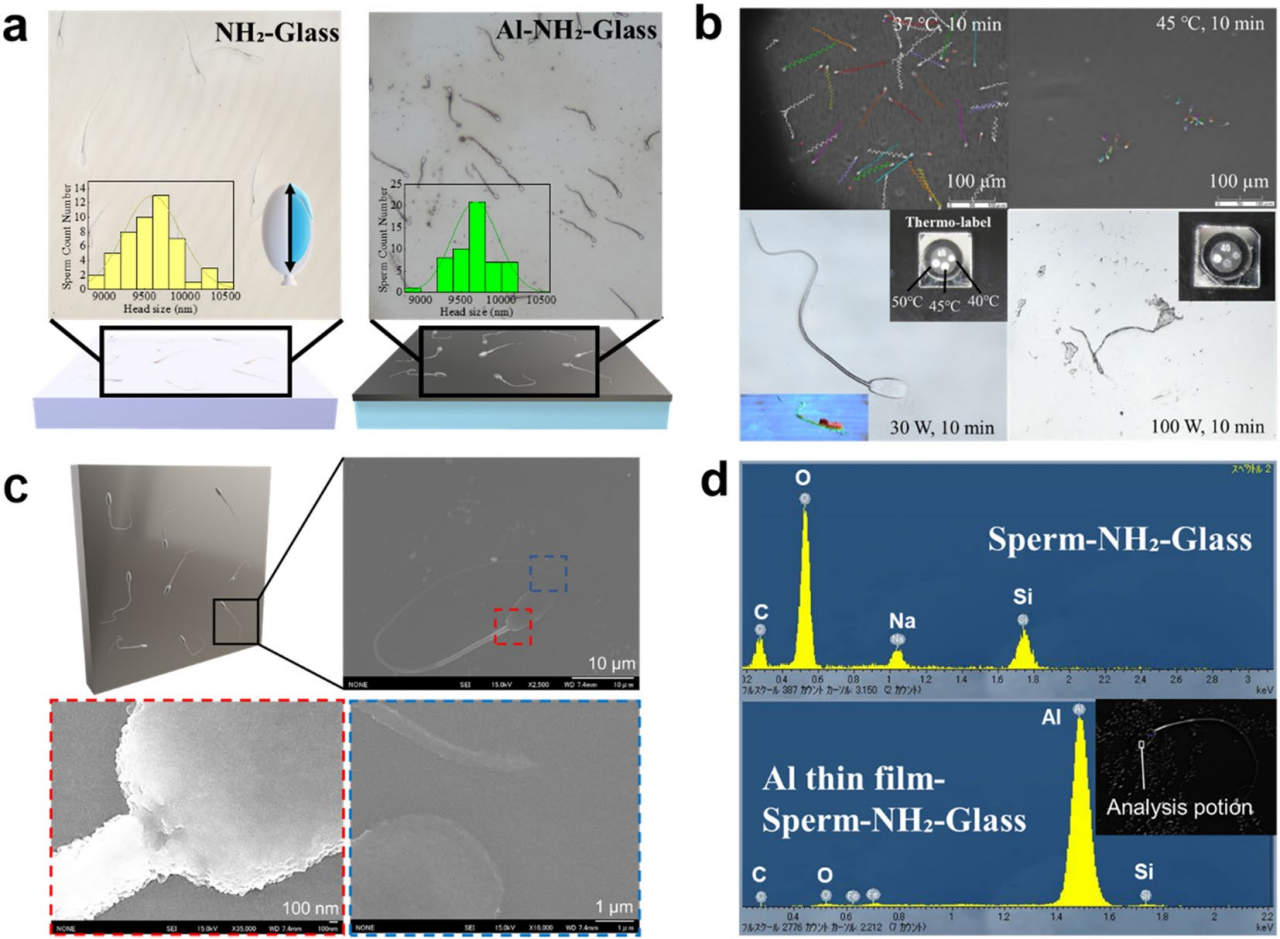
Deposition of Al thin film using non-heat damage technique. (**a**) Glass after sperm adsorption and deposition of Al thin film; laser microscopy imaging was conducted using high magnification. Inset graphs show the result of optical analysis of the sperm head size from tip to neck. (**b**) Motility of spermatozoa was analysed after incubation at 37 °C and 45 °C for 10 min. Films were deposited under temperature conditions conforming to respective results, and the effect of thermal denaturation on sperm shape was observed. (**c**) Scanning electron microscopy images showing sperm from head to tail after Al thin film deposition. (**d**) Elemental analysis of the sperm-glass and sperm heads for Al-sperm-glass.

Furthermore, the sperm was deposited on the Al thin film and then similarly analysed using laser microscopy. There was no significant change in the sperm head size and clear images were observed, suggesting that the formation of the Al thin film did not cause thermal degeneration (Fig. 2a). To confirm reproducibility of this process, another sperm adsorption substrate was prepared, and sperm before and after deposition were observed using a laser microscope (Extended Data Fig. 2). There was no change in sperm surface shape before and after deposition. At least, the protein structure did not change due to thermal denaturation, indicating that the deposition was reproducible. To clarify the effects of heat on the bovine sperm, the motility of the sperm under different temperature conditions was compared and the temperature increase during high-power sputtering was analysed (Fig. 2b). In the motility performance analysis conducted at 37 °C for 10 min, most sperm within the viewing range showed active flagellar motility (Extended Data Table 1).

In contrast, at 45 °C for 10 min, none of the sperm showed flagellar movement, and thermal denaturation of the tail was observed. The optimal deposition temperature of Al that is essential to maintaining the shape of the sperm with active flagellar motility has been shown to be ≤ 40 °C. The film was deposited with a Temperature-label ® attached to the back of the glass. The cathode, which can generate plasma even at a low radio frequency (RF) power of 30 W, deposited the Al thin film without discolouring the Temperature-label ®. Laser microscopy images of the spermatozoa did not reveal any microstructural changes.

Furthermore, the optical height analysis result illustrated in the inset image clearly shows the difference in the nanoscale thickness of the sperm head, midpiece, and tail. Sputtering at 100 W discoloured the label on the back side of the glass at 45 °C. The adsorbed sperm on the substrate showed a change in the surface structure due to thermal denaturation. High vacuum during sputtering is also associated with structural changes, whereas air-drying of sperm has been proven to have little effect on their morphology^18^.

A scanning electron microscope (SEM), which can be used to clearly observe the whole image of the sperm, was used to observe the surface of the Al thin film samples in more detail (Fig. 2c). Fine ridges on the surface and neck were clearly observed in the middle and head of the sperm. The Al thin film, which was shown to be deposited on the sperm surface with high adhesiveness, was analysed and confirmed to be Al using energy-dispersive X-ray spectroscopy (EDS, Fig. 2d). The spectra obtained from the elemental analysis clearly showed the deposition of Al in contrast to the test conducted on the uncoated substrate, which showed no deposition (Extended Data Table 1). In our study, the substrates were pre-treated to become hydrophilic surfaces during the sperm adsorption process, and therefore, the binding and cohesive energies of adatoms are weaker than the cohesive energy to the substrate surface and the resultant layer-by-layer stacking. Thus, interfacial structures of sperm are preserved during our sputter-deposition process.

### Mould fabrication for shape reproduction and observation

**Figure 3:**
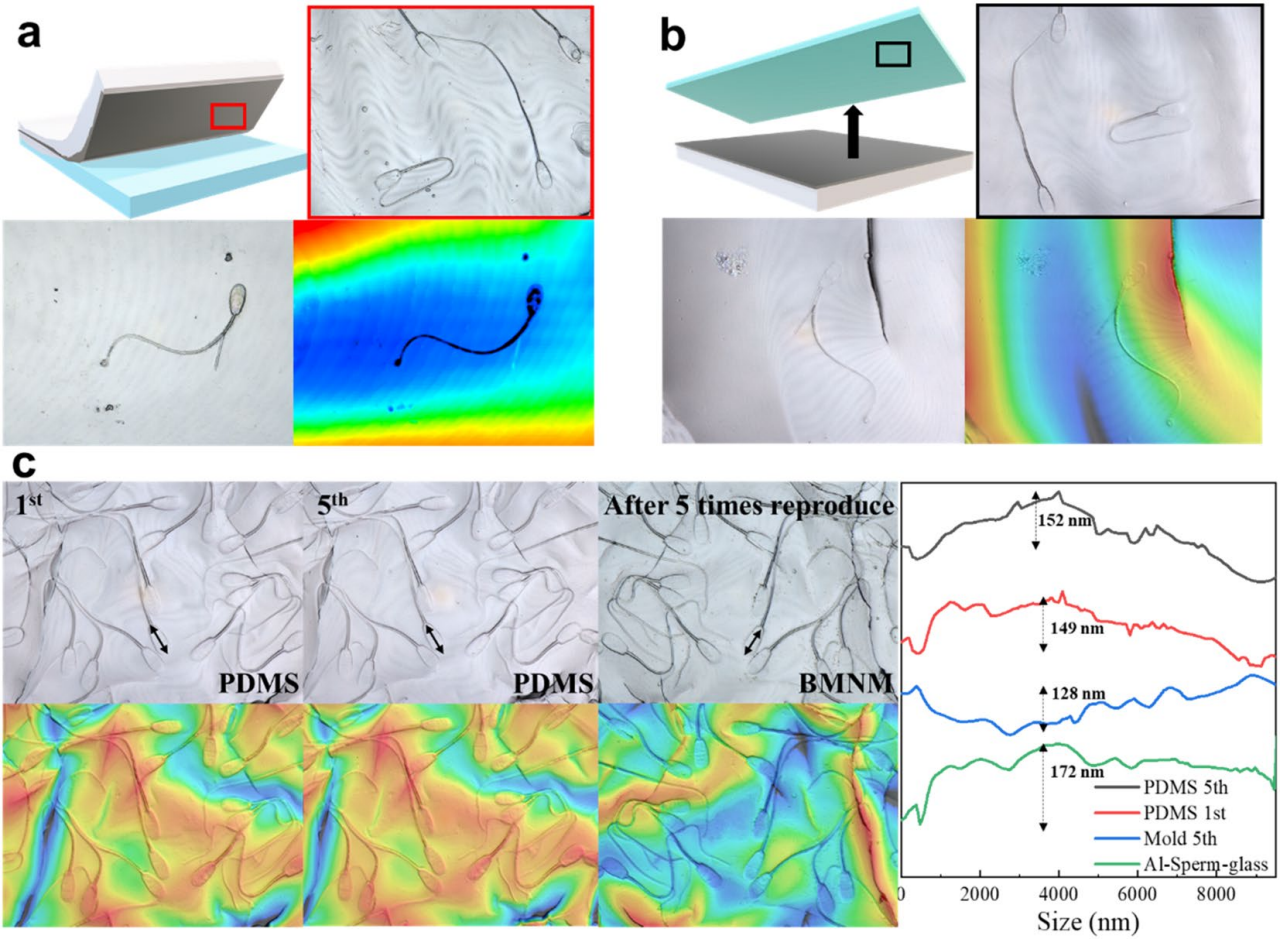
Shape reproduction analysis and observation of fabricated mould. (**a**) Schematic diagram shows stripping method for producing Al thin film from substrate using tapes; the surface of stripped film was analysed using a laser microscope (Red box). Height analysis of sperm with abnormally shaped middle pieces and tails. (**b**) Dimethylpolysiloxane (polydimethylsiloxane, PDMS) was poured into biomimetic metal nano-moulds (BMNM) to reproduce sperm surface shape. PDMS surface separated from the mould was optically analysed using laser microscopy (Black box). Height analysis of sperm with abnormally shaped middle pieces and tails. (**c**) Surface of the first and last PDMS bases; BMNMs were examined using laser microscopy after five replications using the same BMNM. Height of optical head surface was analysed using laser microscopy.

Biomimetic metal nano-moulds (BMNMs) were fabricated using a simple method where the Al film was peeled off the glass using tapes. Optical analysis of the exfoliated Al thin film surface using a laser microscope revealed numerous indentations that were similarly shaped to the sperm (Fig. 3a). The heights of the sperm observed on the BMNM were analysed focusing on those with abnormal shapes of the midpiece and tail.

The optical height analysis suggested that the abnormalsperm shape was clearly concave, suggesting that the deposited Al thin film was formed with a high degree of adhesion to the sperm. The surface morphology was highly preserved even with BMNMs fabricated using a simple procedure. In contrast, the presence of microscale wave-like shapes on the fabricated BMNMs was a problem caused by the tape used for peeling. There are several possible solutions to this problem, such as metal plating of a microscale thickness on the thin films.

The shape of the bovine sperm surfaces was reproduced using PDMS because of its high shape recognition ability^19,20^. The PDMS moulding method was performed by pouring a mixture containing a degassed crosslinker and heating at approximately 70 °C for 4 h. When the moulded PDMS was separated from the mould, inverted sperm with the same shape as the mould were observed on the surface (Fig. 3b). The optical height analysis showed a convex area on PDMS that mimics the sperm shape, and the height analysis of the surrounding area showed a severe height difference. This was likely because the PDMS surface was affected by micro-scale irregularities of the tape surface, which was the base of the mould. The shape was reproduced five times using the same BMNM (Fig. 3c). The PDMS surface analysis using laser microscopy successfully showed that the sperm had the same shape as those in the first and fifth PDMS bases. The surface shape of the mould showed high preservation in the performance evaluation. For the BMNM, the shape of the sperm was clearly observed on the surface after the fifth round of shape reproduction. This observation suggests that the thin Al film did not damage the mould at a temperature of 70 °C, required to rapidly form PDMS.

In addition, height analysis of the same sperm head of each sample using laser microscopy showed a high degree of agreement in the characteristics of irregularities. The same analysis was performed for different sperm after sputtering the Al thin film, and the difference in height was smoother than that of PDMS. This suggests that PDMS reproduced the adhesive surface at the microscale level between the sputtered film and the sperm surface because the pattern of irregularities on the sperm head surface was similar to that on the Al-sperm-glass.

### Detailed structural surface analysis of sperm

**Figure 4:**
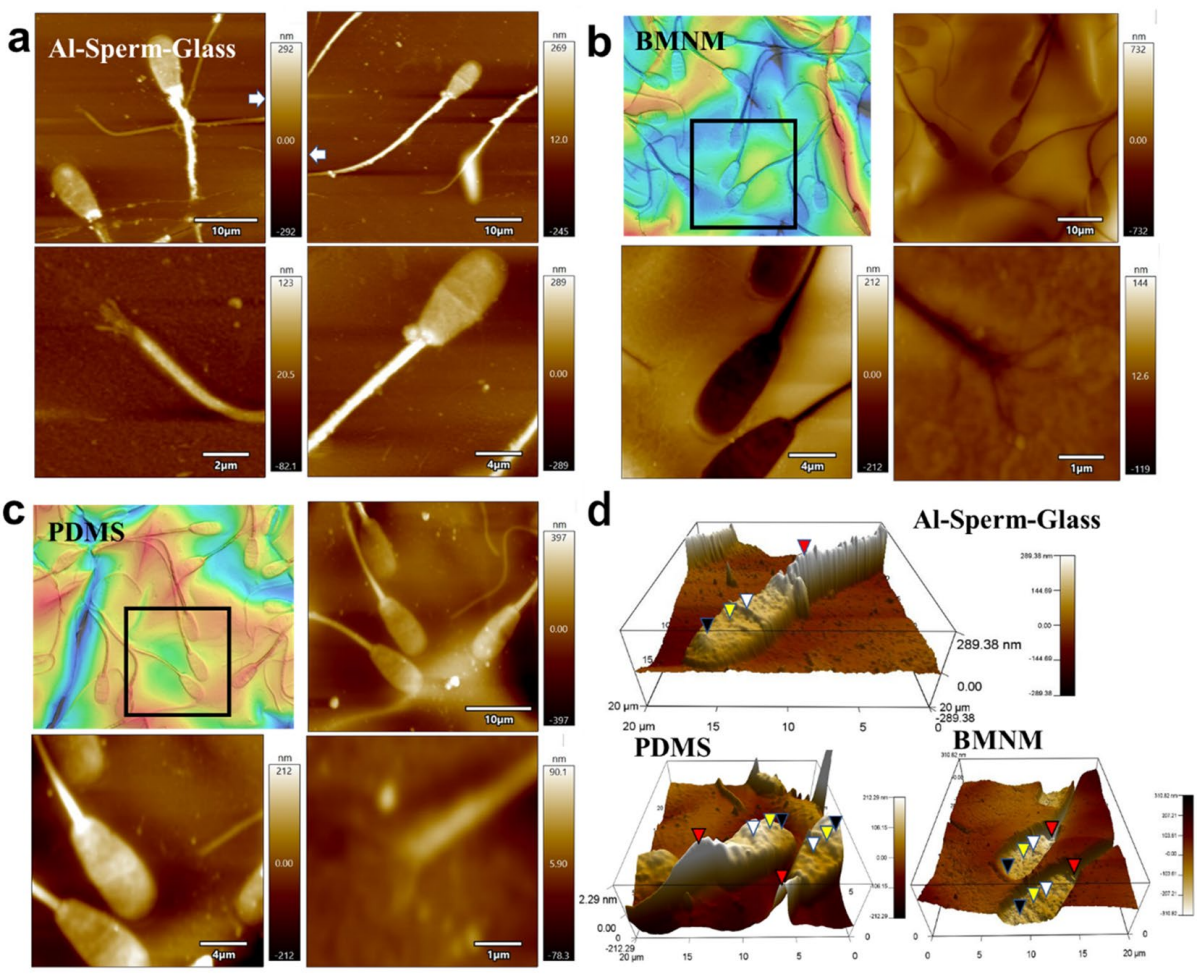
Analysis of various structures of sperm surface. (**a**) Atomic force microscopy analysis of sperm from head to tail after Al thin film deposition. White squares show areas analysed in greater detail. (**b** and **c**) Examination of surface morphology of the sperm stored in biomimetic metal nano-moulds (BMNM) and shape reproduced on dimethylpolysiloxane (polydimethylsiloxane, PDMS) using AFM. Laser microscopy image focusing on sperm shown in black frame. (**d**) Height analysis of AFM image and three-dimensional height mapping. Triangles show microstructure of the sperm head. Black = acrosome, yellow = equatorial segment (EqS), white = necklace, red = midpiece.

The shape of Al-coated sperm was clearly analysed using atomic force microscopy (AFM) for surface characterization (Fig. 4a). A more detailed analysis focusing on the sperm head showed a difference in height towards the tip, similar to the results of the optical height analysis. The increase in height from the head to the midpiece was also consistent with the optical analysis results. By focusing on the tail, it was possible to analyse the fine structure of the tail tip, which was not observed using laser microscopy. At the tip of the tail, fine fibres were separate, indicating that the sperm tail was composed of fibrous tubulin proteins. The normal sperm tail is known to consist of a 9 + 9 + 2 fibre pattern^21,22^. The number of fibres observed in this target was not confirmed, but the related findings suggest that the exposed microstructure maintained its shape after Al sputtering at a biological temperature. AFM has already been established for microbial observation^23^; however, biological temperature sputtering demonstrated superiority in long-term preservation of AFM samples, as it does not require consideration of shape changes due to thermal denaturation.

The BMNM and reproduced PDMS were also analysed using AFM (Fig. 4b and c). The same sperm were analysed from the field of view of the laser microscope described in the previous section. In BMNM, it was possible to analyse the shape of the sperm using laser microscopy. This was evidenced by the microstructure of the exposed tubulin tail. In the PDMS sperm, high-resolution images from the head to tail were successfully captured and examined, as in the Al-sperm-glass, but tubulin at the tip of the tail was not clearly observed. The rubbery nature of PDMS made it difficult to observe the submicron soft parts.

Analysis of the three-dimensional maps of AFM images showed distinct microstructures (Fig. 4d). An equatorial segment, membrane pits, and a necklace in the middle were observed in the heads of the Al sperm, and the height of the midpiece from the head was clear^24^. These structures have already been confirmed by direct observation of the raw adsorbed sperm on the substrate using AFM^25,26^. These microstructures were found on both the BMNM and PDMS that reproduced the shape. This proves that the BMNM preserved the biological surface structures by sputtering with high adhesion at the nanoscale.

## Discussion

We demonstrated the creation of nano/microscale biomimetic moulds with reduced thermal denaturation. The sperm adsorbed in this test was reproduced on the mould in various shapes and states. The fabricated mould was capable of repeated shape reproduction using the PDMS of the sperm. In this study, the moulds were fabricated using heat-resistant tape for simplicity, but more advanced procedures such as metal plating can be used to reproduce moulds using a wider range of materials.

The results of this study show a new relationship between metals and biological samples. The surface structure of biological samples, which is difficult to preserve undamaged for a long period of time, can be permanently preserved using this method^27^. In addition, the imitation of biological samples reproduced from moulds would provide a more appropriate environment for observing microbial samples. However, the material that can be used for shape reproduction depends on the strength of the tape and temperature regime because BMNM executes the transfer process via simple adhesive peeling. The next step includes BMNM made entirely of metal, which could enable us to reproduce biological structures on metallic glasses. The future aim is to create moulds that perfectly mimic the surface shape of biological specimens with superhydrophobic structures and antifouling properties. For industrial applications, BMNM would enable mass production in biomimicry, which has a large potential for producing multifunctional surface structures. On the contrary, the biothermal deposition method can be useful for the film deposition of not only biomaterial surfaces but also other thermally sensitive materials. Our low-temperature deposition method can be applied to some thermal-sensitive semiconductor devices or polymer substrates, although an appropriate surface condition of substrate is required for using our deposition method.

## Methods

### Chemical agents

(3-Aminopropyl)triethoxysilane (APTMS) was purchased from Sigma-Aldrich. Ethanol, super dehydrated toluene, and phosphate-buffered saline (PBS) buffer were purchased from Wako (Osaka, Japan). PDMS (SILPOT 184) was purchased from Toray, Ltd. (Tokyo, Japan). The bovine sperm samples used were separated from commercially available cryopreserved semen straws from Japanese black bulls. Sperm-Tyrode's albumin lactate pyruvate buffer (SP-TALP) was purchased from Caisson Laboratories, Inc. (Smithfield, UT, USA).

### Amino-coating of glass

A micro glass slide (Matsunami, Osaka, Japan) was functionalized by coating it with APTES in super dehydrated toluene. In the first step of the modification procedure, all purchased glass slides were rinsed in ethanol and APTES was then added to super dehydrated toluene to a concentration of 2%, which was used as the amino group-modifying solution. The washed glass was placed in this solution for 2 min with both sides immersed. Then, the washed glass was immersed in pure water and super dehydrated toluene for 10 s, and the process was repeated twice.

Finally, the amino group-modified glass was immersed in ethanol, air-dried, and stored. The success of the surface modification was determined based on the difference in the contact angle between the unmodified and modified glass. Specifically, the contact angle was calculated by capturing the height and width of a 2 µL water droplet dropped on the substrate using a charge-coupled device camera and substituting the values into the following Eq. ^28^.

$$\theta = 2\tan^{ - 1} \frac{2a}{b}$$

Amino group modification was performed on the glass to improve the protein adsorption capacity and wettability of the substrate. The contact angle of the amino group-modified glass treated for a short time showed a significant change compared with that of the unmodified glass (Extended Data Fig. 3).

### Bovine sperm sorting

Bovine sperm were used as the biological sample in this study. A large amount of foreign matter is present in semen and a previously reported sorting procedure was used to isolate sperm from the frozen bovine semen^16^. A microfluidic channel with three chambers, fourteen microchannels, and a crescent-shaped diffuser was used to control the counter-fluid flow. The sperm were separated through the microchannel using their ability to resist flow, which is only exhibited by those with normal motility. The flow was controlled for 30 min and the separated sperm solution was used as the sample. The sperm were observed during and after separation using an optical microscope (Eclipse Ti, Nikon, Tokyo, Japan). Sperm motility and images after separation were analysed using a computer-assisted sperm analysis system (SMAS, Ditect Co., Tokyo, Japan). All procedures were conducted at 37 °C to avoid damaging the sperm using a microscope (Diaphot 300, Nikon, Tokyo, Japan). The buffer solution used for sperm isolation contained SP-TALP adjusted to pH 7.

### Glass absorption

The adsorption of sperm onto the amino group-modified glass was achieved based on the movement of sperm in solution and the adsorption force on the glass. The separated sperm solution was incubated at 37 °C and the amino group-modified glass was placed on the bottom of a Petri dish containing the solution. The Petri dishes were incubated for 2 h while maintaining the temperature and the glass was then removed and washed with ultrapure water. The surface of the glass was observed using an optical microscope to confirm the sperm were adsorbed.

### Al thin film formation on glass substrate

The Al thin films were formed on the bovine sperm using a magnetic mirror-type magnetron cathode (M3C); the details of the device structure are reported in Ref 29.

The plasma particles were confined between a pair of strong magnetic fields due to a phenomenon called the magnetic mirror confinement effect. The magnetic mirrors prevented the cross-field diffusion of charged particles, thereby reducing heat shock to the sample surface by secondary electrons emitted from the Al target surface, achieving biothermal sputtering. Sputtering was performed for 10 min at 0.13 Pa (Ar, 10 sccm) with an RF input power of 30 W. The distance between the Al target (99.99% purity) and sample surface was 50 mm. In Al vapor evaporation, the substrate temperature increases up to 60 °C under similar experimental condition with the distance between the evaporation source and irradiated object, deposition time, and gas pressure (Extended Data Fig. 4). Although vapor evaporation is a well-known technique for the pre-treatment of insulating material in SEM measurement, it is not suitable for biothermal coating because the substrate temperature rises by about 60 °C.

### Transfer of Al thin film from glass

The Al thin film was transferred by peeling it off from the glass substrate using a double-sided tape that is heat resistant to up to 250 °C (Chukoh, Tokyo, Japan) follow the Ref 30. The film surface exhibited micro-scale irregularities and the tape which was bonded to the thin film adhered by applying physical pressure. The Al thin film was transferred to the tape side when the tape was peeled off from the glass substrate.

### Shape reproduction of sperm mould using PDMS

PDMS was mixed with the cross-linker at a ratio of 10:1, thoroughly stirred, and the mixture was then degassed using an evaporator. The degassed mixture was poured into a mould and heated at 70 °C for 4 h to polymerize PDMS. The solidified PDMS was physically peeled off the mould and used for subsequent experiments.

### Analysis of samples

All laser microscopic analyses were performed using a VK-X3000 (KYENCE) JSM-9100F (JEOL), which was also used for detailed structural analysis by SEM. Elemental analysis of the substrate surface was performed accurately using EDX (TEAM™ EDS System, HITACHI). Surface roughness analysis was also performed using AFM (MFP-3D Origin + , Oxford Instruments).

## Supplementary Information

Supplementary Information.

## Data Availability

The data that support the findings of the current study are available from the corresponding author upon reasonable request.
