# Supplementary material for: Metal deposition and shape reproduction at biological temperatures on cell-level samples
Source: Sci Rep. 2022 Aug 3;12:13328. doi: 10.1038/s41598-022-17562-9 (PMC9349294; doi:10.1038/s41598-022-17562-9)

**Metal deposition and shape reproduction at biological temperatures on cell-level samples**

Kenshin Takemura^1,＊^, Taisei Motomura^1^, Wataru Iwasaki, Naoki Matsuda

Sensing System Research Centre, The National Institute of Advanced Industrial Science and Technology (AIST), 807-1 Shuku-Machi, Tosu, Saga 841-0052, Japan

^1^These authors contributed equally to this work.

^＊^=Corresponding Author

Extended Data Figure 1. Difference in amount of sperm adsorbed depending on functionalization of substrate was observed using optical microscopy


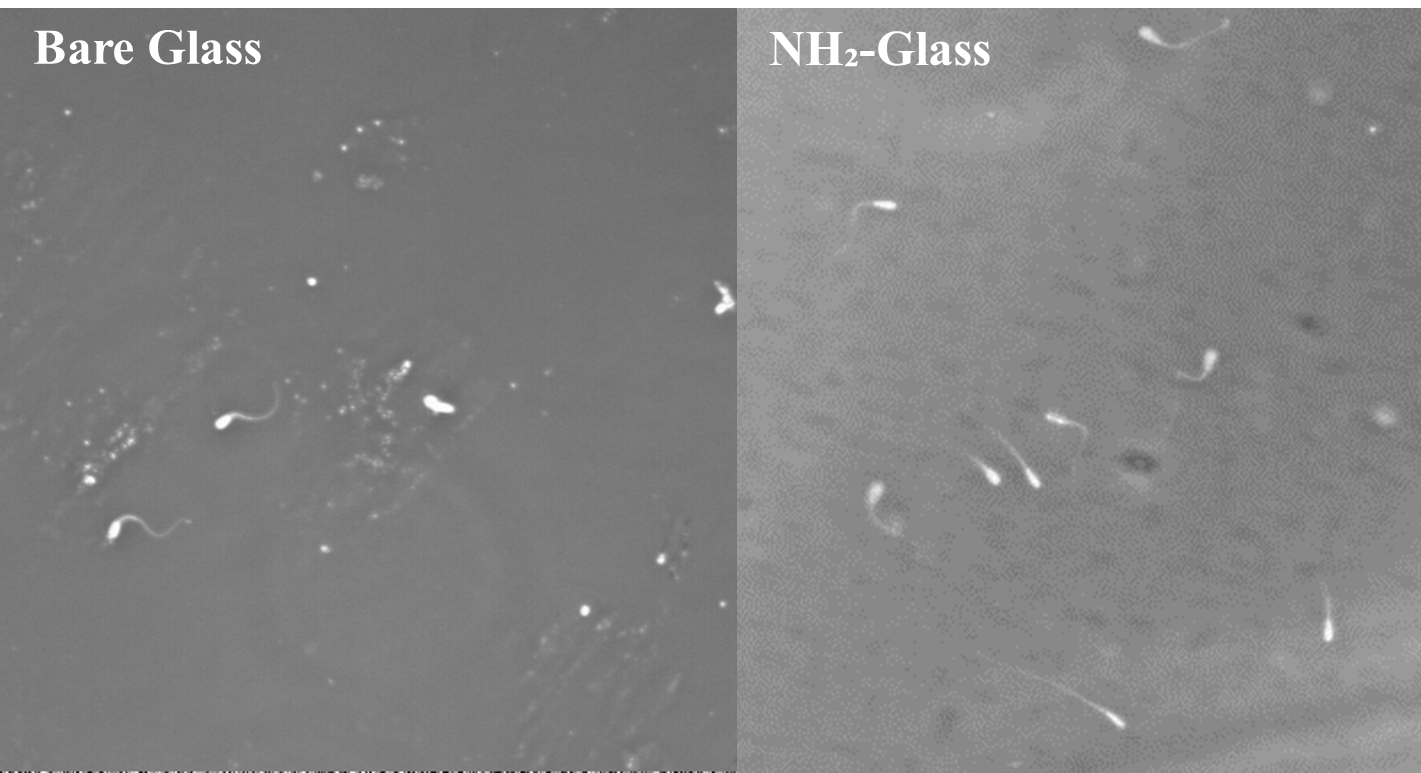


Extended Data Table 1. Difference in amount of sperm adsorbed depending on functionalization of substrate observed using optical microscopy


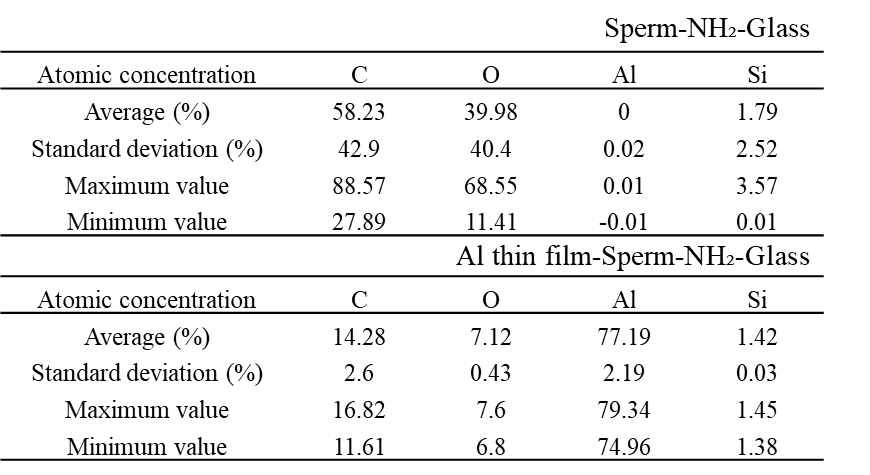


Extended Data Figure 2. Laser microscope images of sperm adsorbed on a glass substrate before and after deposition.


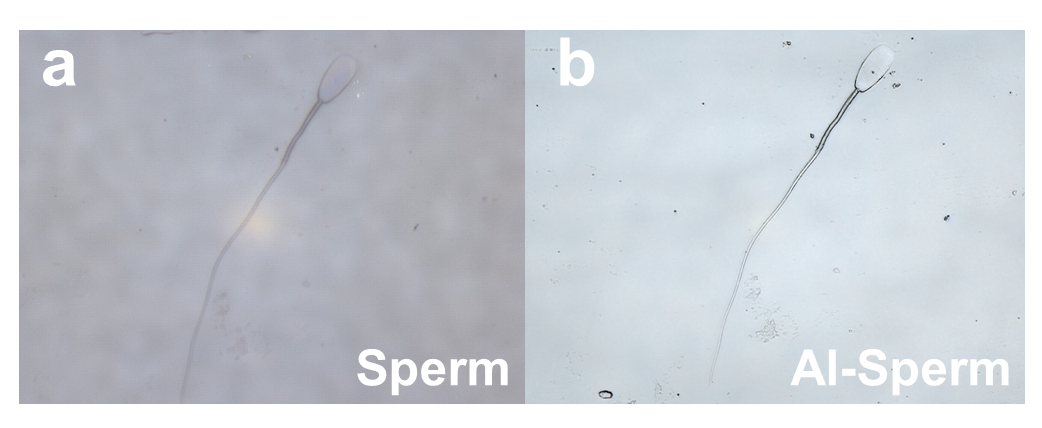


Extended Data Figure 3. Contact angle measurement results for unfunctionalized and amino group-modified glass


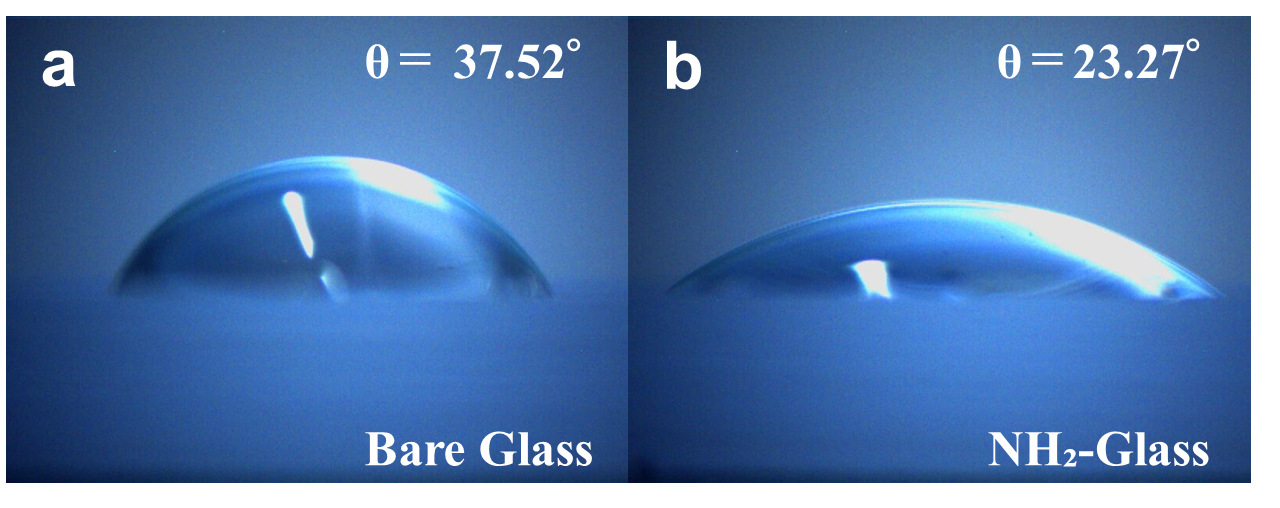


Extended Data Figure 4. Results of the aluminium vapor deposition test on thermolabel by vapor deposition. a) Arrangement of thermolabel stuck on substrate, b) thermolabel before deposition, c) thermolabel after deposition, d) thermolabel after the removal of surface protection tape (labels that have undergone thermal discoloration are highlighted in white boxes).


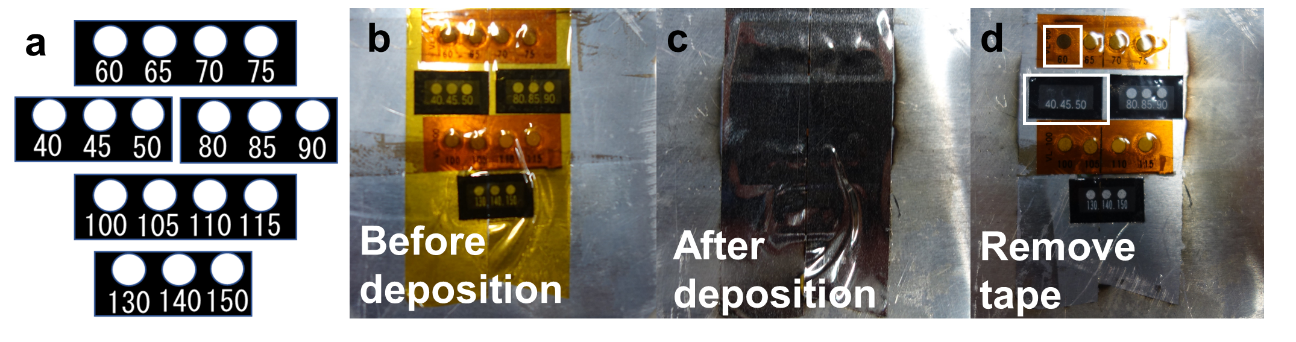

Supplement: Supplementary file 1 — Supplementary Information. [file 41598_2022_17562_MOESM1_ESM.docx]
